# ASO Author Reflections: Intraoperative Radiation Therapy (IORT): Will It Survive in the USA?

**DOI:** 10.1245/s10434-023-13982-7

**Published:** 2023-07-22

**Authors:** Melvin J. Silverstein, Brian Kim, Shane Lloyd, Peter Chen, Kevin Lin

**Affiliations:** 1 Department of Surgery, Hoag Memorial Hospital Presbyterian, Newport Beach, CA USA; 2 Department of Surgery, Keck School of Medicine, University of Southern California, Los Angeles, CA USA; 3 Department of Radiation Oncology, Hoag Memorial Hospital Presbyterian, Newport Beach, CA USA

Intraoperative radiation therapy (IORT) is a single dose of radiation therapy given intraoperatively during the lumpectomy procedure in the hope that it will be the only adjuvant radiation therapy required. All local treatment is completed during a single operative procedure. There is 100% compliance and minimal damage to surrounding tissue. This all sounds great. However, IORT has been around for more than 20 years and there are only about 100 centers in the USA offering the procedure. And now, its future is threatened.

In this issue of the *Annals*, we report the results of an IORT registry trial that we began in 2010: 1600 cases with a median follow-up of 63 months.^1^ The 5-year local recurrence rate was 5.18%. If whole breast radiation therapy (WBRT) had been used instead of IORT, we would have expected only about 1% of patients to recur in this time period. So, how can we justify a recurrence rate five times higher? We would say that 5% is still a low number, a number lower than what we accepted when we first started doing breast conservation in the 1980s and 1990s. Moreover, during our trial, we have learned how to pick better patients for IORT. One of our low-risk subgroups has a recurrence rate of less than 2%.

IORT has been used for more than 40,000 patients worldwide. It was slow to find its way into the USA because of the lack of long-term data and poor reimbursement, but now, two prospective randomized trials have shown acceptably low long-term local recurrence rates with no difference in overall or breast-cancer-specific survival.^2,3^ With these data, one would think breast IORT would be poised to expand in the USA, but it is not. We will tell you why shortly.

## Past

One of us (M.J.S.) was surgically trained in the late 1960s. At that time, breast cancer was not understood as the nuanced disease we know today. There was no screening. No small early cancers. Most patients presented with palpable node positive disease. Most were treated with the Halsted radical mastectomy. The breast, the pectoralis muscles, and all axillary nodes were removed. Patients with positive nodes received postoperative radiation therapy. Patients with node-negative disease often received postoperative radiation therapy if the index lesion was medial or central. The only chemotherapies available were 5FU and methotrexate. Patients did poorly oncologically, physically, and psychologically. This operation, which was not like any mastectomy we perform today, left a deformed, maimed, and emotionally scarred woman who was thankful just to be alive and perhaps saved from a premature breast cancer death. She was often miserable because of her deformity. You could count her ribs on the mastectomy side when you looked at her. Arm pain and arm swelling were common and so were recurrences. Most surgeons didn’t think twice about the mutilation they had caused. They were not trained to think that it was important. They were trained to cure cancer by removing it and the healthy tissue around it. They were interested in technical results. Did the skin graft take? How many nodes were found?

There was a man in the 1960s who did care about mutilation: Umberto Veronesi, MD, Director of the Italian National Cancer Institute. He fought to start a trial comparing radical mastectomy with segmental resection plus whole breast irradiation. He was successful at getting the trial done and in 1981, he published the 5-year results, ushering in the possibility of breast conservation. Survival was equal in both arms of the trial.^4^ The study was published in the *New England Journal of Medicine* and discussed on the first page of *The New York Times*. Bernard Fisher, MD, et al. would follow with similar equivalent survival results in 1985.^5^ However, breast conservation would not really be widely used in the USA for another 10 years. Old ideas die hard.

## Present

Today, breast conservation is the state-of-the-art, the standard of care. Today, we hate a poor cosmetic result, and we teach young surgeons to care about how the patient looks and feels. We care so much that we developed oncoplastic surgery, a combination of oncologic tumor-removing destructive surgery and plastic reconstructive surgery. Today, it’s possible for a patient to look better after breast cancer surgery than before. This was inconceivable in the past.

In a conversation with Professor Veronesi before he died, he explained how his interest in IORT developed. Although breast conservation was proven to yield equal survival when compared with mastectomy, it required post-excisional whole breast radiation therapy. This took 5–6 weeks at that time. Many women, although they were excellent candidates for breast conservation, did not have the time for everyday treatment or did not live near enough to a radiation therapy center to make breast conservation a viable option. So regardless of how favorable their disease was, they underwent mastectomy to cure it. To give those women a chance at breast conservation, intraoperative radiation therapy with electrons was trialed in Milan.

Fast forward to today. Two major groups^2,3^ have done prospective randomized IORT trials with acceptable long-term local recurrence rates but with minimal acceptance of the technique in the USA. During the last 20 years, while IORT was being developed, breast radiation therapy advanced, and there are now 3–4 weeks accelerated courses of WBRT. There are also 5-day courses and local recurrence rates are only about 1–2% at 5 years. Although local recurrence rates are higher for IORT, overall survival and breast-cancer-specific survival are the same whether we do IORT, WBRT, or even mastectomy.

The American Society for Radiation Oncology (ASTRO) recently posted a draft of practice guidelines for partial breast irradiation, asking for public input (ASTRO.org). In the section on IORT, they referred to the long-term results of the prospective randomized TARGIT and ELIOT trials, trials that have allowed much of Europe to make IORT a standard option. ASTRO’s conclusion was not to recommend IORT. Their reason was that the 15-year recurrence rate in the ELIOT Trial was too high (12.6% for IORT versus 2.4% for WBRT). The TARGIT 5-year recurrence rate was only 2.11%, but that did not seem to impact their decision. At this writing, we have not seen the final version. While nothing ASTRO says is binding, they are a powerful organization. Their position not to recommend IORT could very well end the use of this technique in the USA and erase 20 years of progress and the benefits that have been accrued to tens of thousands of patients.

So where does that leave us? If ASTRO maintains its preliminary position that they will no longer support IORT in any patients, the technique may be doomed in the USA. A better approach would be to support it in selected low-risk patients. ELIOT patients that fit the ASTRO suitable category had a 1.5% rate of local recurrence at 5 years, pointing out the importance of patient selection. Our hope is that ASTRO will recognize the need and the benefit of maintaining IORT as an option in selected low-risk populations. If ASTRO does not support IORT, insurance companies will no longer pay for IORT, and we will regress back to the place where Professor Veronesi found himself in the mid 1990s as he searched for an alternative to WBRT.

Is there any reason to accept a higher local recurrence rate? We think there is. The list of potential IORT benefits is long. There is 100% compliance. The same cannot be said for WBRT.

IORT is less expensive (one dose versus many). IORT offers a better quality of life: less pain, cosmetically superior (no breast shrinkage), no travel back and forth to the radiation therapy center, and single-dose treatment. It’s green: less gas, less pollution, less travel. It can be done without extra time off from work. There is no need for childcare. It’s better for patients with challenging conditions such as Parkinson’s, multiple sclerosis, wheelchair bound, autism, etc. It’s better for the homeless and incarcerated. It’s better for non-English speaking patients. There’s less hospital exposure, so less risk of coronavirus disease 2019 (COVID-19) or other air-borne respiratory illnesses. There is less radiation dose to the skin, heart, and lungs. There are no table-top weight limits for patients with obesity and more. We believe that in some patients, these benefits may offset the increase of local recurrence. We believe the patient should be allowed to hear the arguments for and against IORT and make her own decision.

## Future

If IORT is not seen as a viable option by radiation oncology societies, all of its benefits will be gone in the USA. If reason and careful interpretation of data prevail, IORT will remain a viable option for the properly chosen patient.
